# Investigation of African swine fever outbreaks in pigs outside the controlled areas of South Africa, 2012–2017

**DOI:** 10.4102/jsava.v91i0.1997

**Published:** 2020-07-16

**Authors:** Leana Janse van Rensburg, Juanita van Heerden, Mary-Louise Penrith, Livio E. Heath, Thapelo Rametse, Eric M.C. Etter

**Affiliations:** 1 Department of Production Animal Studies, Faculty of Veterinary Science, University of Pretoria, Pretoria, South Africa; 2 Directorate Animal Health, Department of Agriculture, Forestry and Fisheries of the Republic of South Africa, Pretoria, South Africa; 3 Onderstepoort Veterinary Research, Agricultural Research Council, Onderstepoort, South Africa; 4 Department of Veterinary Tropical Diseases, Faculty of Veterinary Sciences, University of Pretoria, Pretoria, South Africa; 5 TADScientific, Pretoria, South Africa; 6 CIRAD, UMR Animal, Santé, Territoires, Risque et Ecosystèmes (ASTRE), Montpellier, France; 7 ASTRE, University of Montpellier, CIRAD, INRA, Montpellier, France

**Keywords:** African swine fever, domestic cycle, biosecurity, disease control, auctions, swill

## Abstract

South Africa historically experienced sporadic African swine fever (ASF) outbreaks in domestic pigs in the northern parts of the country. This was subsequently indicated to be because of spillover from the sylvatic cycle of ASF between warthog and tampans (soft ticks) in the area. South Africa declared this area an ASF-controlled area in 1935, and the area is still controlled in terms of the *Animal Diseases Act, 1984* (Act 35 of 1984). Two main epidemics of ASF in domestic pigs were identified outside of the South African ASF-controlled area. The first occurred in 2012 with outbreaks in Gauteng and Mpumalanga provinces, and the second occurred in 2016–2017 with outbreaks in the North West, Free State and Northern Cape provinces. These were the first ASF epidemics in South Africa associated with transmission of the disease via a domestic cycle. This study found that the spread of ASF in these epidemics was mainly via auctions, swill feeding and scavenging. These three aspects need to be addressed in terms of awareness and education on the disease including implementation of biosecurity measures in order to prevent future ASF outbreaks in South Africa. Specific biosecurity measures should be implemented in the semi-commercial sector to prevent ASF-infected pigs and pig products from being moved to naïve pigs and therefore spreading the disease.

## Introduction

African swine fever (ASF) in domestic pigs is a devastating disease and one of the main limitations for pig production in sub-Saharan Africa (Fasina et al. 2012; Penrith 2009). This haemorrhagic disease usually has a very high mortality rate, and currently no vaccine is available as a means of prevention, nor is there any means of treatment (Bastos, Fasina & King 2014; Costard et al. 2009; Penrith & Vosloo 2009). These factors discourage investment in the pig sector (Penrith et al. 2013). The disease has detrimental effects on the socio-economic situation of farmers: for subsistence farmers, particularly in terms of food security, and for commercial farmers, the disease can cause enormous economic losses (Babalobi et al. 2007).

As pork is a reasonably priced source of protein, pig production has increased worldwide (Orr & Shen 2006). This, combined with the ASF virus (ASFV) stability and persistence in meat products that have not been processed sufficiently to inactivate the virus, has led to the dissemination of the disease along transport and trading routes (Bastos et al. 2014). This potential for rapid spread of the disease makes it imperative that the disease be promptly diagnosed and control measures enforced to prevent the disastrous economic and social consequences of outbreaks (Chenais et al. 2017; Etter et al. 2011).

Historically, three different epidemiological cycles were described for ASF: the natural sylvatic cycle, the domestic pig-tick cycle and the domestic cycle (Bastos et al. 2014). Recently, a fourth cycle has been described in Europe, the wild boar-habitat cycle, which is different from the sylvatic cycle involving warthogs (Chenais et al. 2018).

In the natural sylvatic cycle, circulation of the virus is maintained by transmission between the common warthog (*Phacochoerus africanus*) and argasid ticks (*Ornithodoros moubata* complex), allowing the disease to remain endemic in Southern and Eastern Africa (Chenais et al. 2018; Jori et al. 2013; Penrith et al. 2013; Sánchez-Vizcaíno et al. 2015; Wilkinson 1986). The disease is thought to have evolved in this cycle, with the ticks inhabiting warthog burrows, feeding on their blood and transmitting the virus at the same time (Plowright, Parker & Peirce 1969; Thomson 1985).

The domestic cycle is defined by the maintenance of viral circulation by transmission of the disease between domestic pigs without the involvement of an arthropod biological vector (Penrith et al. 2013). Although outbreaks can be initiated when infected ticks feed on a domestic pig, subsequent spread of the virus within domestic pig populations depends largely, if not entirely, on the horizontal transmission of the virus between pigs. Transmission can occur through direct contact between infectious and susceptible pigs, consumption by susceptible pigs of contaminated meat products and contact with fomites (Penrith & Vosloo 2009; Penrith et al. 2013). This cycle depends on a constant supply of susceptible pigs exposed to the virus (Penrith et al. 2013).

The first description of an ASF outbreak in South Africa was in 1928 in pigs close to Modimolle in Limpopo Province and was followed by several outbreaks for a few years in that vicinity (Penrith 2013). Since 1928, there have been frequent reports of ASF in the northern part of the country (De Kock, Robinson & Keppel 1940; Magadla et al. 2016). In 1935, South Africa declared a controlled area for ASF where the disease is endemic because of the sylvatic cycle; this included parts of the Limpopo, Mpumalanga, North West and KwaZulu-Natal provinces (Magadla et al. 2016; Penrith 2013). The disease is still controlled in South Africa in terms of the *Animal Diseases Act, 1984* (Act 35 of 1984).

The first outbreak of ASF reported outside of the ASF-controlled area of South Africa was in 1996, just outside the controlled area in the Warmbaths region (around the town of Bela-Bela) in Limpopo Province (Magadla et al. 2016). This was an isolated incident, suspected to have been caused by the illegal movement of domestic pigs from the ASF-controlled area and did not spread beyond the index farm (Penrith & Vosloo 2009). Since then, two epidemic episodes occurred outside the controlled area, one in 2012 and one between 2016 and 2017 (Department of Agriculture, Forestry and Fisheries [DAFF] 2018). There is very little published information available on these large-scale outbreaks of ASF in the previously ASF-free zone of South Africa. Phylogenetic studies on the viruses involved are ongoing and the results will be published separately when they have been completed. The purpose of this study is to provide a brief epidemiological description of the 2012 and 2016–2017 ASF outbreaks to identify factors in the pig production systems that may have contributed to the spread and maintenance of infection and that can be subjected to further epidemiological analysis to determine their relative importance. Considering the zoning system in place in South Africa for the control of ASF, it is crucial for policy-makers, the pig industry and the international community to understand the factors that contributed to the occurrence of these outbreaks and spread of the disease outside of the controlled area of South Africa.

## Research methods and design

This study investigated ASF outbreaks in domestic pigs, exclusively outside of the ASF-controlled area of South Africa, from 2012 to 2017. During the period under study, provincial veterinary officials reported an ASF outbreak to the DAFF for each epidemiological unit where ASF had been confirmed, which was subsequently reported to the World Organisation for Animal Health (OIE) by DAFF. These epidemiological units were each a group of pigs that had the same likelihood of exposure and consisted of pigs on the same farm or free-roaming pigs in the same communal area.

Primary epidemiological information was collated from official veterinary disease reports submitted for each of the events by the provincial state officials responsible for the areas in which the outbreaks occurred. This was supplemented by utilising the follow-up reports from the provincial veterinary services and personal communication with officials that had been involved in the outbreak control and eradication. The minutes of the meetings held by the ASF Veterinary Operational Committee, as well as communication between the South African Pork Producers’ Organisation (SAPPO) and DAFF relating to the ASF outbreaks, were also utilised for supplementary information.

Maps used throughout this article were created using ArcGIS^®^ software (Esri^1^) and timelines were created using Microsoft Excel 2013^®^.

### Diagnosis

For this study, we analysed the results from diagnostic tests performed during the studied ASF outbreaks at the Agricultural Research Council – Onderstepoort Veterinary Research, Transboundary Animal Diseases laboratory (ARC-OVRTAD), which is the Africa-based OIE Reference laboratory for ASF testing. Organ and blood samples collected during the outbreak investigations were tested to detect the presence of the ASFV genome using the real-time polymerase chain reaction (PCR) to confirm outbreaks. When serum samples were submitted, enzyme-linked immunosorbent assay (ELISA) was performed with the commercially available blocking ELISA, which uses a monoclonal antibody (Mab) specific for VP72 ASFV protein, manufactured by Ingenasa (Ingezim PPA Compac K3, Ingenasa, Madrid, Spain).

### Ethical consideration

Ethical approval was obtained for this research as part of a doctoral project from the Research Ethics Committee of the Faculty of Veterinary Science, University of Pretoria (project number REC011-19).

## Results

### 2012 epidemic

**FIGURE 1:**
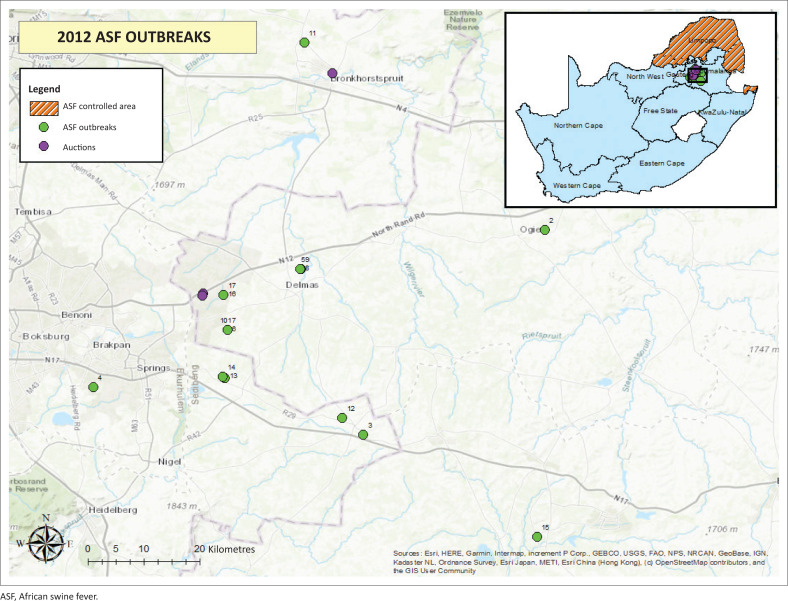
Map of locations involved during the 2012 African swine fever epidemic.

During the ASF epidemic of 2012, 17 outbreaks were reported outside of the South African ASF-controlled area: six outbreaks in Gauteng Province and 11 outbreaks in Mpumalanga Province (Figure 1). The first case was discovered at an abattoir in Gauteng Province on 04 January 2012, when on post-mortem inspection a meat inspector and local veterinarian found septicaemic carcasses at an abattoir in the Lesedi municipality (Geertsma, Mpofu & Walters 2012). The lesions raised a suspicion of either ASF or classical swine fever. Samples taken from the carcasses were submitted to the ARC-OVR TAD for laboratory confirmation. Following laboratory confirmation of ASF, the remaining pigs were euthanised and all carcasses were destroyed. Quarantine of the abattoir was lifted following disinfection with a chlorine-based chemical.

The farm of origin of the diseased pigs was found to be about 40 km from the abattoir located in the Victor Khanye Municipality, Mpumalanga Province. Upon investigation, it was found that the owner had bought pigs at an auction in Sundra, Mpumalanga, at the end of November/beginning of December 2011. Both the farm of origin and the auction were situated outside of the ASF-controlled area of South Africa. Soon after purchasing these pigs, they had started showing clinical signs of illness, which the owner had presumed to be because of respiratory disease. The owner treated the pigs with antibiotics, but as this produced no improvement in the health of the pigs or the number of mortalities, the owner decided to send the remaining pigs for slaughter towards the end of December 2011.

The provincial veterinary officials investigated the auctioneers’ records and obtained the addresses and contact information of the owners of pigs traded at the auctions of November/December 2011. Following intensive trace backward and forward activities on the part of the veterinary services, three auctions were implicated in the spread of the disease, two in Sundra (Mpumalanga) and one in Bronkhorstspruit (Gauteng).

In total, it was found that in 14 of the 17 ASF outbreaks, owners had traded pigs at auctions. Swill feeding or access to infectious material through scavenging may have caused the remaining three outbreaks. The details of these reported outbreaks are captured in Table 1.

**TABLE 1:** Summary of the 2012 African swine fever epidemic outside the South African controlled area.

Outbreak report no.	Outbreak report date	Province	Comments	Number of dead pigs†	Number of pigs culled†	Total
1	2012-01-13	Mpumalanga	Bought pigs at auction in Mpumalanga; discovered on post-mortem at abattoir in Gauteng	37	52	89
2	2012-01-18	Mpumalanga	Bought pigs at auction in Mpumalanga; traced via auction records	34	2	36
3	2012-01-18	Gauteng	Bought pigs at auction in Mpumalanga; traced via auction records	42	88	130
4	2012-01-19	Gauteng	Bought pigs at auction in Mpumalanga	73	44	117
5	2012-01-20	Mpumalanga	Bought pigs at auction in Mpumalanga; traced via auction records	34	15	49
6	2012-01-20	Mpumalanga	Bought pigs at auction in Mpumalanga; traced via auction records	2	92	94
7	2012-01-20	Mpumalanga	Bought pigs at auction in Mpumalanga; traced via auction records	1	5	6
8	2012-01-20	Mpumalanga	Bought pigs at auction in Mpumalanga; traced via auction records	0	8	8
9	2012-01-20	Mpumalanga	Bought pigs at auction in Mpumalanga; traced via auction records.	0	12	12
10	2012-01-23	Mpumalanga	Bought pigs at auction in Mpumalanga; traced via auction records	15	32	47
11	2012-01-25	Gauteng	Bought pigs at auction in Gauteng	10	0	10
12	2012-01-26	Gauteng	Pigs free roaming and scavenge for food	7	2	9
13	2012-02-03	Gauteng	Bought pigs at auction in Mpumalanga	25	14	39
14	2012-02-03	Gauteng	Dead pig brought from neighbouring infected property and owner practises swill feeding	4	17	21
15	2012-02-03	Mpumalanga	Bought pigs at auction in Mpumalanga; traced via auction records	1	6	7
16	2012-02-28	Mpumalanga	Outbreak in informal settlement where pigs are not formally housed and scavenge for food	196	603	799
17	2012-03-06	Mpumalanga	Bought pigs at auction in Mpumalanga	44	104	148
**Total**				**525**	**1096**	**1621**

† , Approximate numbers – where numbers differ from the World Organisation for Animal Health (OIE) reports; this is because of the information only becoming available after the OIE report had been made.

The original source of the epidemic according to provincial and national veterinary services may have been because of the illegal movement of pigs from a farm within the ASF controlled area located in the Lephalale Municipality, Limpopo. Upon investigation by Limpopo Veterinary officials, it was discovered that a farm had supplied pigs to auctioneers outside of the controlled area. According to the workers, pigs had started dying on the farm prior to pigs being sold to the auction, and the pigs had had direct contact with warthogs. Unfortunately, no more pigs were left on this farm to confirm the presence of ASFV.

From when the first ASF was confirmed in January 2012, a Veterinary Operational Committee was formed consisting of members from DAFF, the Provincial Veterinary Services of Gauteng and Mpumalanga as well as SAPPO. This committee coordinated control measures for the ASF outbreaks, which included movement controls in a 3 km radius around reported outbreaks, patrols by the stock theft unit of the South African Police Service, disinfection of auction premises and backward as well as forward tracing from the auction records. Auctions were closed until 60 days after disinfection of the premises. Basic biosecurity measures were recommended, which would prevent spread of ASF and could be speedily implemented, without laborious and costly infrastructure changes. This included confining pigs (SAPPO assisted with feed for confined ex-free-roaming pigs), not feeding swill, not bringing in pigs of unknown health status and not allowing unauthorised people contact with the pig herd. Awareness amongst farmers was raised on the clinical signs of ASF in order to promote reporting of suspicious clinical signs; SAPPO assisted with this initiative that included holding farmer’s days and distribution of pamphlets.

**FIGURE 2:**
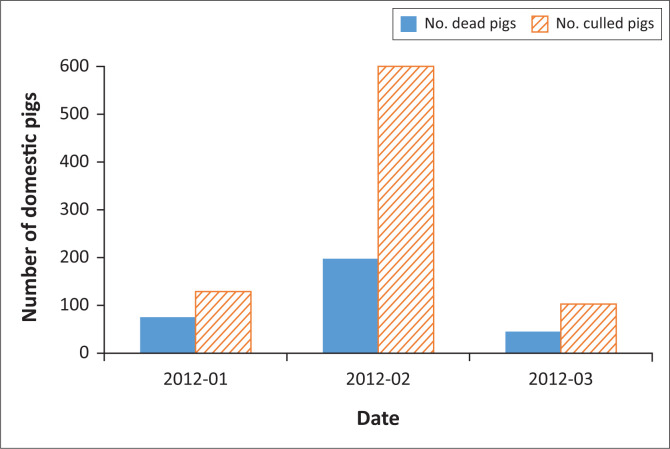
Number of domestic pigs affected per month during the 2012 African swine fever epidemic.

These described outbreaks in 2012 were eradicated by selective culling. This entailed quarantining premises confirmed to be infected with ASF and euthanising the remaining pigs. The carcasses were destroyed and the premises disinfected. Farmers had signed contracts with SAPPO who paid incentives for the remaining pigs on the premises to be culled. The pigs were culled by stunning with a captive bolt, followed by an overdose of pentobarbitone intra-thoracically (Geertsma et al. 2012). Representatives from the Society for Prevention of Cruelty to Animals were present for the culling. The carcasses were transported by trucks, lined with leak-proof plastic, to a secure landfill site in order to prevent environmental contamination, disposed of by deep burial, and covered with lime. In total, approximately 525 pigs died and 1096 were culled during the 2012 ASF epidemic, which was reported from January 2012 to March 2012 (Figure 2). All outbreaks were declared resolved by 22 May 2012.

### 2016–2017 epidemic

**FIGURE 3:**
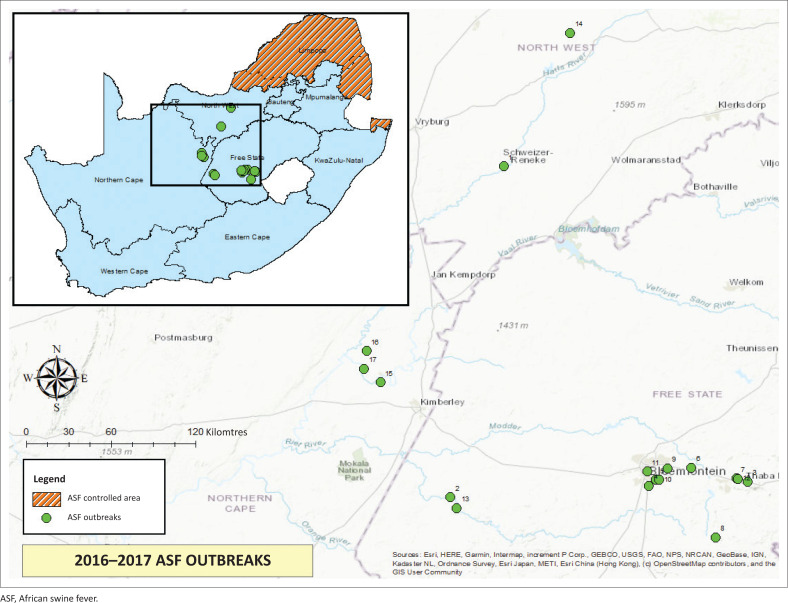
Map of 2016–2017 African swine fever epidemic outbreaks.

During the ASF epidemic of 2016–2017, another 17 outbreaks were reported outside of the ASF controlled area and spanned three provinces: two in North-West Province, 12 in Free State Province and three in Northern Cape Province (Figure 3).

The first two outbreaks in this epidemic occurred approximately simultaneously in May 2016: one in the North West Province and one in the Free State Province. The first outbreak in the North West Province was reported in a communal township area where various farmers owned free-roaming pigs. At the same time, the Free State veterinary services had been called to a farm on 26 May 2016 where 250 free-range pigs had died over a period of 2 weeks. The farm was placed under quarantine and samples were confirmed positive for ASF. The farmer reported that he had sent pigs to an auction in Bloemfontein at the end of March as well as supplied slaughtered pigs to a client in Koffiefontein commonage in April before mortalities had been experienced.

Trace forward and backward activities ensued as well as increased surveillance for the disease, which found further outbreaks in Free State, North West and Northern Cape. The details of these reported outbreaks are shown in Table 2. The initial source of the 2016–2017 epidemic was not confirmed, but it may be that once introduced the virus spread either via swill, intentional feeding or because of scavenging of free-roaming pigs.

**TABLE 2:** Summary of the 2016–2017 African swine fever epidemic outside the South African controlled area.

Outbreak report no.	Outbreak report date	Province	Comments	Number of dead pigs†	Number of pigs culled†	Total
1	2016-05-26	North West	Free-roaming pigs which scavenge, occasional swill feeding. Group of 15–20 communal small scale farmers. May have moved pigs to Bloemfontein area	623	420	1043
2	2016-06-07	Free State	Pigs kept free ranging and scavenge in area with swill feeding also practised. Sold pigs at an auction in Bloemfontein area	250	30	280
3	2016-06-27	Free State	Bought pigs at auction in Bloemfontein area; sold pigs locally and at auction	257	9	266
4	2016-06-27	Free State	Pigs bought locally and at auction; pigs may scavenge	79	25	104
5	2016-06-27	Free State	None	100	20	120
6	2016-07-05	Free State	Pigs bought on auction in Bloemfontein area	23	9	32
7	2016-07-18	Free State	Communal farmers with free-roaming pigs that may scavenge	3	13	16
8	2016-07-26	Free State	Subsistence farmers who visited relatives in other outbreak areas in the time period just before the outbreak. Farmers practise swill feeding. The pigs were loosely contained, with some able to escape	5	0	5
9	2016-08-11	Free State	None	3	6	9
10	2016-08-29	Free State	None	50	66	116
11	2016-09-16	Free State	Communal farmers with free-roaming pigs that may have had direct contact with other free-roaming pigs (closest outbreak was 2 km away)	25	371	396
12	2016-09-16	Free State	Communal farmers with free-roaming pigs that may scavenge	5	6	11
13	2016-11-01	North West	Small scale farmer that feeds swill from a lodge on the property, which borders a game reserve	41	6	47
14	2016-12-09	Free State	None	30	18	48
15	2017-02-23	Northern Cape	Communal farmers with free-roaming pigs that may scavenge and are occasionally fed swill, within an enclosed community	65	9	74
16	2017-06-14	Northern Cape	Farm with pigs kept in a camp around the homestead that are fed swill	42	1	43
17	2017-07-05	Northern Cape	Communal farmers with free-roaming pigs that may scavenge	195	2	197
**Total**				**1796**	**1011**	**2807**

† , Approximate numbers – where numbers differ from World Organisation for Animal Health (OIE) reports; this is because of the information only becoming available after the OIE report had been made.

As was the case in the 2012 outbreaks, a selective culling policy was followed for the control of the outbreaks of the 2016–2017 epidemic. This entailed culling all the pigs kept on enclosed properties, but this was more complicated in communal areas where it was difficult to clearly define epidemiological units. In these cases, pigs that were infected or confirmed to be in contact with infected pigs were culled. Other groups of pigs in the area for which there was no compelling evidence of contact with infected animals were first monitored for any signs of disease followed by testing before a decision on whether to proceed with culling the animals was taken. Farmers were served with quarantine notices when ASF was confirmed. Each province’s veterinary services approached the outbreaks in their own manner, but in most cases, the Provincial Veterinary Services formed a Joint Operation Committee consisting of representatives from the Municipality, Department of Health, Police and Traffic Police, and the Department of Social Development, who worked together with the Disaster Management Committee to address the outbreaks. In some of the cases, the Department of Social Development assisted farmers affected by the ASF outbreaks with donations and together the committee organised equipment and burial sites to reduce the risk of contamination and exhumation of the pig carcasses. Some carcasses and confiscated pork products were burnt; other carcasses were buried and covered with lime. Movement control was implemented for pigs and pig products from affected properties with the assistance from traffic police and local police assisting with random patrols checking for these items.

**FIGURE 4:**
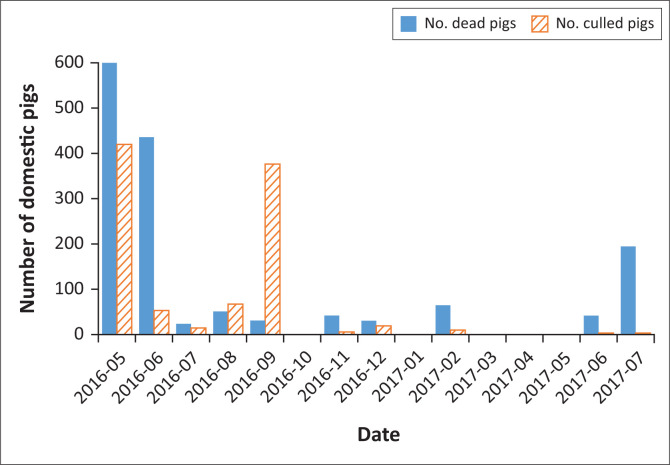
Number of domestic pigs affected per month during the 2016–2017 African swine fever epidemic.

The pigs that were culled were shot or stunned/killed by captive bolt and/or euthanised with pentobarbital. Vehicles, equipment and protective clothing used were washed and disinfected. During the 2016–2017 epidemic, about 1796 pigs died and about 1011 were culled (Figure 4).

In the cases where farmers chose to restock, following culling and disinfection, sentinel pigs were placed and quarantine was only lifted once the sentinel pigs remained clinically healthy and tested serologically negative for ASF after a 3-month period had elapsed. In these cases, none of the sentinel pigs had contracted ASF. In some communal areas, where not all pigs in the wider area had been culled, there was increased clinical surveillance as well as serological surveillance, which indicated that the disease had not established itself in the domestic pig population generally. This epidemic was assumed to have started with the first reported outbreak in May 2016 and continued until the last reported outbreak in July 2017, thus lasting 15 months. After the last reported outbreak, surveillance activities continued for 5 months, after which all outbreaks of this epidemic outside the ASF-controlled area were declared resolved in December 2017.

## Discussion

This study describes the first two ASF epidemics outside the ASF-controlled area of South Africa. These epidemics were the first occurrences of widespread ASF outbreaks constituting a domestic cycle in South Africa. People were found to play the main part in the spread of ASF by moving pigs, pig products and other objects (including the people themselves) contaminated with the infectious virus, which is corroborated by the findings of other studies (Fasina et al. 2015).

During the 2012 ASF outbreaks, the disease was spread by trade of pigs at auctions and to a lesser degree, by the feeding of swill or by scavenging. This was the first widespread outbreak of ASF outside of the ASF-controlled area, as well as the first series of outbreaks of ASF that resulted from a domestic cycle, even though the original source of the outbreaks according to back-tracing was likely spillover from the sylvatic cycle within the ASF endemic area of South Africa. This is supported by the findings of Fasina et al. (2015), who confirmed various links for pig movements between Limpopo, Mpumalanga and Gauteng.

South Africa has very strict import requirements for live pigs, pig genetic material and pork products and does not allow the import of live pigs or pig genetic material from countries with ASF (DAFF Animal Health: Import/Export Policy Unit – personal communication). Legally imported animals, genetic material or pig products were thus not the likely source of ASFV introduction.

Although the exact source of the 2016–2017 epidemic was not determined, it may be that the initial introduction(s) were followed by inter-farm and inter-location transmissions, primarily by swill feeding and scavenging. Although Regulation 24 of the *Animal Diseases Act, 1984* (Act 35 of 1984) prohibits the feeding of swill unless boiled for 60 min or sterilised by means of another efficient method, this has been found difficult to enforce, especially in resource-poor communities. At the two index farms (North West and Free State), both reported that workers may have brought pig products into the area and disposed of this material in such a manner that the pigs may have had access to these products. This study emphasises the potential risk of swill feeding and indicates that further studies are needed to study swill feed dynamics in terms of where and how swill feeding is practised in South Africa.

With an increased emphasis on food security, combined with the fact that with a free-roaming pig keeping system investment is minimal, small-scale piggeries are on the increase in Southern Africa (Penrith et al. 2013). Statistics South Africa (2016) found in its Community Survey on Agricultural Households that the number of households keeping pigs in South Africa increased from 112 678 in 2011 to 210 504 in 2016. Of these households, 192 257 (91%) kept between 1 and 10 pigs. It follows that an increasing informal pig population would supply more susceptible pigs, which may, in the absence of biosecurity and movement controls, present a greater risk for the occurrence of a domestic cycle of ASF (Penrith et al. 2013). With these informally kept pigs, more ASF risk factors are added by the marketing systems, because of lack of organisation, use of auctions, lack of abattoirs and proper meat inspection, especially for pigs coming from smallholder farms (Penrith et al. 2013). Another factor that can increase the occurrence of ASF in domestic pigs is the expansion of communal residential areas, with the keeping of free-roaming domestic pigs as an inexpensive protein source. Free-roaming pigs are predisposed to coming into contact with various sources of infective material (Penrith & Vosloo 2009).

The potential for rapid spread of this disease was seen in the outbreaks of 2012, as it was found that 172 farms and about 10 374 pigs had primary or secondary contact with the index farm (Fasina et al. 2015). It follows that eradication of this disease in domestic pig populations can be difficult, expensive, laborious and may take a considerable time, as shown by the eradication of ASF following earlier outbreaks from the 1950s in European and South American countries (Penrith & Vosloo 2009). As neither treatment nor vaccine is available for ASF, control must start with preventive measures such as biosecurity and disease awareness and having an early warning system. Once an outbreak is reported, control is based on quarantine, disinfection and culling (Bastos et al. 2014; Beltrán-Alcrudo et al. 2017). During the outbreaks under study, it was fortunate that the SAPPO understood the added value of a public–private partnership with government and was willing to incentivise culling of pigs on infected premises. This resulted in limiting the spread of these outbreaks as well as speedy eradication of the domestic cycle. This example of good public–private partnership should be emulated by organised pig industries elsewhere.

The spread of ASFV during these two epidemics was linked to pig and pig product movements and could have been prevented by good biosecurity practices. Fasina et al. (2015) found that in the Limpopo Province, South Africa, which is mostly part of the ASF-controlled area, there were informal pig movements and trade networks, and that producers were prepared to travel up to 400 km for markets, which would indicate that these emerging smallholders should be a focus for prevention strategies.

Another risk that needs to be addressed is that a great proportion of pig slaughter was performed informally for local consumption, which means that ante- and post-mortem inspection are unlikely to have been performed. The latter can be an important step in identifying ASF, especially where farmers would want to salvage some monetary value when pigs start dying (Penrith et al. 2013). This was demonstrated with the initial diagnosis of the 2012 ASF epidemic being made on post-mortem inspection at an abattoir.

The role of auctions in the 2012 outbreaks indicates that government, industry and auctioneers need to cooperate to compile a plan to prevent disease spread by looking at basic biosecurity requirements, good practices, including improving traceability and supervision that would promote animal health throughout South Africa. Industry organisations such as the National Animal Health Forum of South Africa are ideally situated to facilitate such initiatives.

To date there have been no ASF outbreaks in commercial piggeries in South Africa where basic biosecurity measures have been implemented. The ASF outbreaks in the described epidemics occurred mostly in communal pigs and some smallholder farms with little or no biosecurity. Several commercial piggeries in South Africa have subscribed to the officially endorsed biosecure compartment system. This system was based on the OIE described concept of physical and managerial biosecurity practices to prevent entry of disease to farms in order to maintain a subpopulation of animals of a specific health status (World Organisation for Animal Health 2018). The biosecurity measures implemented by pig compartments conform to a biosecurity plan approved by the veterinary services and include physical barriers such as warthog-proof fencing and solid housing, management practices such as avoidance of swill feeding and showering in with the use of farm-only protective clothing. Only pigs certified to originate from another officially approved compartment are allowed to enter a compartment. These measures have proven effective in South Africa. According to Fasina et al. (2012), a biosecurity framework should be economically justifiable, easy to integrate into the established farm practices and assure sufficient support from workers on the farm.

Swill feeding, in cases where pork from informal slaughter facilities is distributed between families and neighbours, should be addressed. This is important considering that the virus can be maintained in the meat from infected pigs for long periods. Rather than pursuing an enforcement policy for this important biosecurity measure, it is proposed that awareness and education of the semi-commercial and informal pig sector should be prioritised in order to promote compliance. This could be developed into a system where cooperatives are formed in these communities, where they could get access to animal health care as well as work together to access better marketing opportunities.

## Conclusion

This study found that the three main means of spread of ASF in these two epidemics (2012 and 2016–2017) were the trade of pigs at auctions, the feeding of swill and free-roaming pigs that scavenge for food. These three aspects need to be addressed in terms of awareness, education and implementation of risk mitigation measures in order to prevent future ASF outbreaks in South Africa. Specific biosecurity measures should be implemented in the semi-commercial sector to prevent ASF. These biosecurity measures need to be low-cost and developed specifically for these types of farmers. In the end, the success of prevention of ASF does not only rely on the government veterinary services of a country, as unscrupulous people may circumvent legislation for profit, but also rely on stakeholder support and participation from all spheres of the pig industry.

## Footnotes

1 ArcGIS® and ArcMap™ are the intellectual property of Esri and are used herein under license. Copyright © Esri. All rights reserved. www.esri.com.
